# Testing the decoy effect to increase interest in colorectal cancer screening

**DOI:** 10.1371/journal.pone.0213668

**Published:** 2019-03-26

**Authors:** Sandro Tiziano Stoffel, Jiahong Yang, Ivo Vlaev, Christian von Wagner

**Affiliations:** 1 Research Department of Behavioural Science and Health, University College London, London, United Kingdom; 2 University of Warwick, Warwick Business School, Coventry, United Kingdom; London School of Hygiene and Tropical Medicine, UNITED KINGDOM

## Abstract

Literature on consumer choice has demonstrated that the inclusion of an inferior alternative choice (decoy) can increase interest in a target product or action. In two online studies, we tested the impact of decoys on the probability of previous non-intenders to have a screening test which could significantly lower their chances of dying of colorectal cancer. We find that the presence of a decoy increased the probability to choose screening at the target hospital (over no screening) from 39% to 54% and 37% to 59% depending on how many hospital attributes were communicated and how strongly the decoy was dominated by the target. We also show that the presence of the decoy was associated with lower levels of reported decisional complexity while not undermining information seeking and knowledge acquisition. These findings offer a ‘proof of principle’ that decoys have the potential to increase screening uptake without negatively influencing informed choice.

## Introduction

Human decision-making can be influenced or ‘nudged’ in a predicted direction through careful manipulation of choice setting [1]. One classic example of how individual preferences can be influenced is the decoy effect, whereby the introduction of a less attractive alternative (i.e. a decoy) into a choice set increases the probability of the more attractive target or action being chosen [2;3].

The fundamental idea underlying the decoy effect is that designing the decoy in such a way that it is only inferior to the target but not the competitor, it improves the perceived attractiveness of the target, thus increasing its likelihood to be chosen. Three meta-analyses suggest that decoys can increase the likelihood by 12 to 18% [4–6]. The decoy effect has been consistently demonstrated in various choice settings such as product selection [2;7;8], gambling [2;9], employee selection [10;11], political elections [12;13] hand hygiene [14] and medical decision-making [15–17]. While most of them consisted of hypothetical non-incentivised choice exercises, two showed the decoy effect in field experiments [7;14].

In our current study, we tested whether the decoy effect could be used to influence medical treatment options. Specifically, we investigated if offering individuals the possibility to conduct a colorectal cancer (CRC) screening test at an alternative, less convenient, medical centre could increase interest in having the test at the standard site. CRC is the fourth most common cancer and the second leading cause of cancer-related death in the UK [18]. As early diagnosis of CRC through screening dramatically reduces incidence and mortality of the disease, in England, the NHS offers Bowel Scope Screening (BSS), also known as flexible sigmoidoscopy, as a one-off test for men and women aged 55 [19]. However, uptake for this invasive test is low, especially among low-income and minority groups [20]. Thus novel strategies such as nudges could be applied to increase people’s acceptance of evidence-based cancer screening programs [21].

### The current research

We set out to test whether adding a decoy in the context of BSS increases people’s interest in having the test. Specifically, we presented individuals who initially expressed little or no interest in participating in the BSS with detailed information about the test including information about the hospital in which it would be carried out. We chose to test the decoy among disinclined people to minimise ceiling and social desirability effects often associated with self-reported intention measures [22] and to simulate a targeted intervention aimed at non-attenders who are in most need of an effective behavioural intervention.

For the purpose of creating the decoy we manipulated information about the length of time it would take to travel to the hospital (travel time) and the length of time people would have to wait for their appointment once they had arrived at the hospital (waiting time), as previous research has identified both travel and waiting time as potential barriers to attending appointments [23–25]. Specifically, we chose to create a decoy using waiting and travel time of the screening hospital as they are relatively similar in terms of their underlying metric.

In Study 1 we created a decoy by manipulating either travel or waiting time to test whether the addition of a decoy screening hospital (with longer travel or waiting time than the target hosptital) would increase the likelihood of choosing the target hospital. In addition to hospital choice, we also expected that the decoy would reduce the cognitive burden associated with decision-making by allowing people to use a ‘dominance heuristic’ which stipulates that individuals may find it easier to choose the target as it, but not the non-screening, dominates the decoy [26;27].

Study 2 then varied decoy strength by manipulating two hospital attributes simultaneously to create different levels of decoy strength [2;28]. In addition, we also investigated the impact of offering a decoy on information seeking to gain a better understanding of how offering a decoy alternative would facilitate or undermine people’s ability to make an informed choice about screening to address ethical concerns that nudging may undermine people’s ability to make an informed choice based on knowledge of the harms and benefits of cancer screening [29–31].

## Study 1

Study 1 tested whether the principle of the decoy effect applies to a complex and unfamiliar decision that people face when invited for BSS.

### Method

The protocols for the pilot study and both Study 1 and Study 2 received ethics approval from University College London’s Research Ethics Committee (approval number 13537/001).

#### Procedure

**Fig 1:**
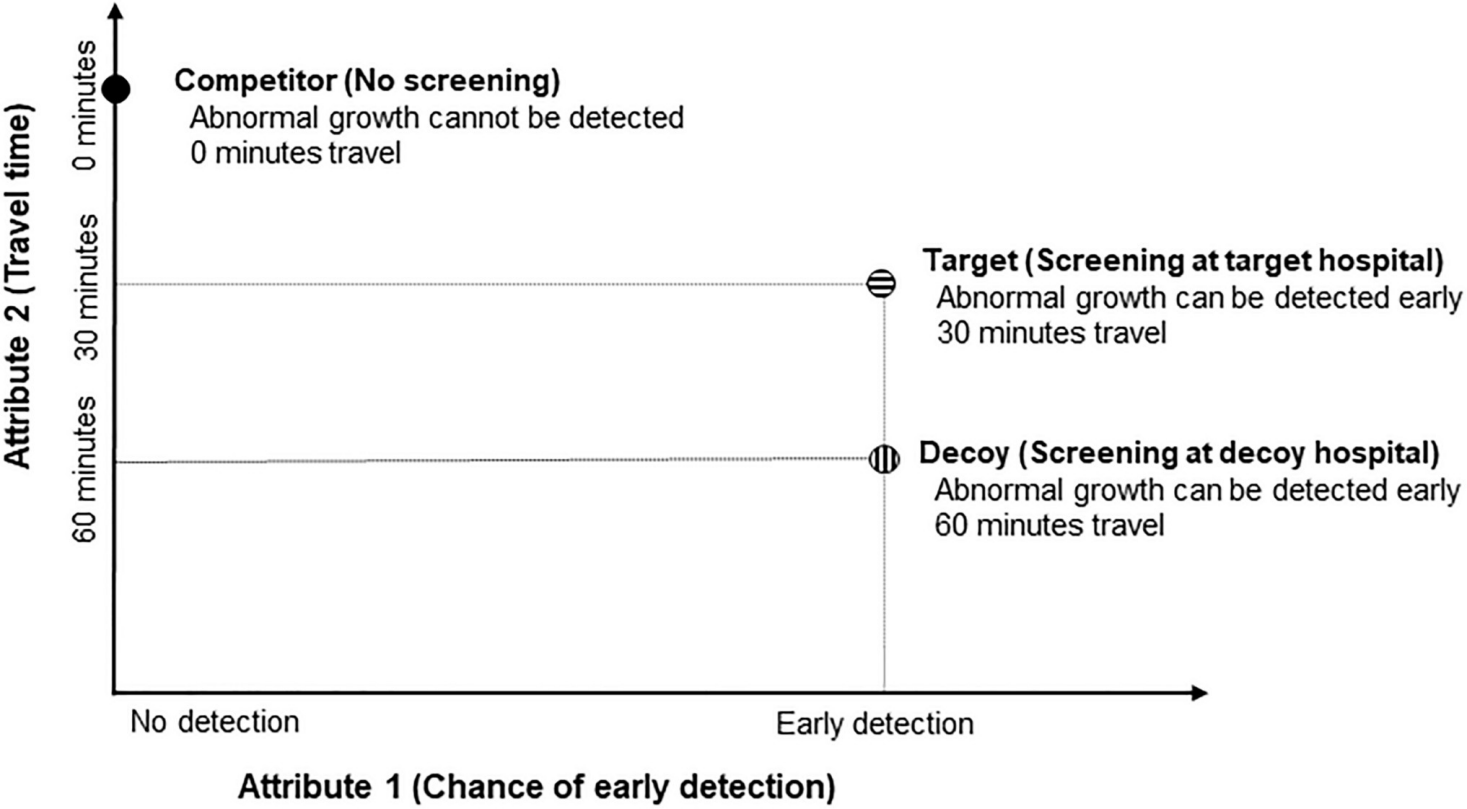
Implementation of the decoy effect in Study 1.

In order to test the effect of adding a decoy to the choice set, we identified attribute levels for travel and waiting times in a preliminary study with 118 respondents (see S1 Table). Levels were selected according to the following criteria: Firstly, the decoy attribute levels should be perceived as inferior than the target attribute levels. This assumption was confirmed in a preference task which presented respondents with two levels: 30 minutes travel time (reflecting typical values from previous data [32] vs. 60 minutes travel time Fig 1).

Secondly, we had to identify corresponding attribute levels for waiting time. In order to derive levels that were equivalent to those for travel time we presented respondents with a series of binary choices between two hospitals that differed in travel and waiting time. These choices resembled a discount rate experiment (see S1 Protocol for sample questions [33;34] and established that the corresponding values for waiting time were approximately 45 minutes for the target and 90 minutes for the decoy hospital.

**Fig 2:**
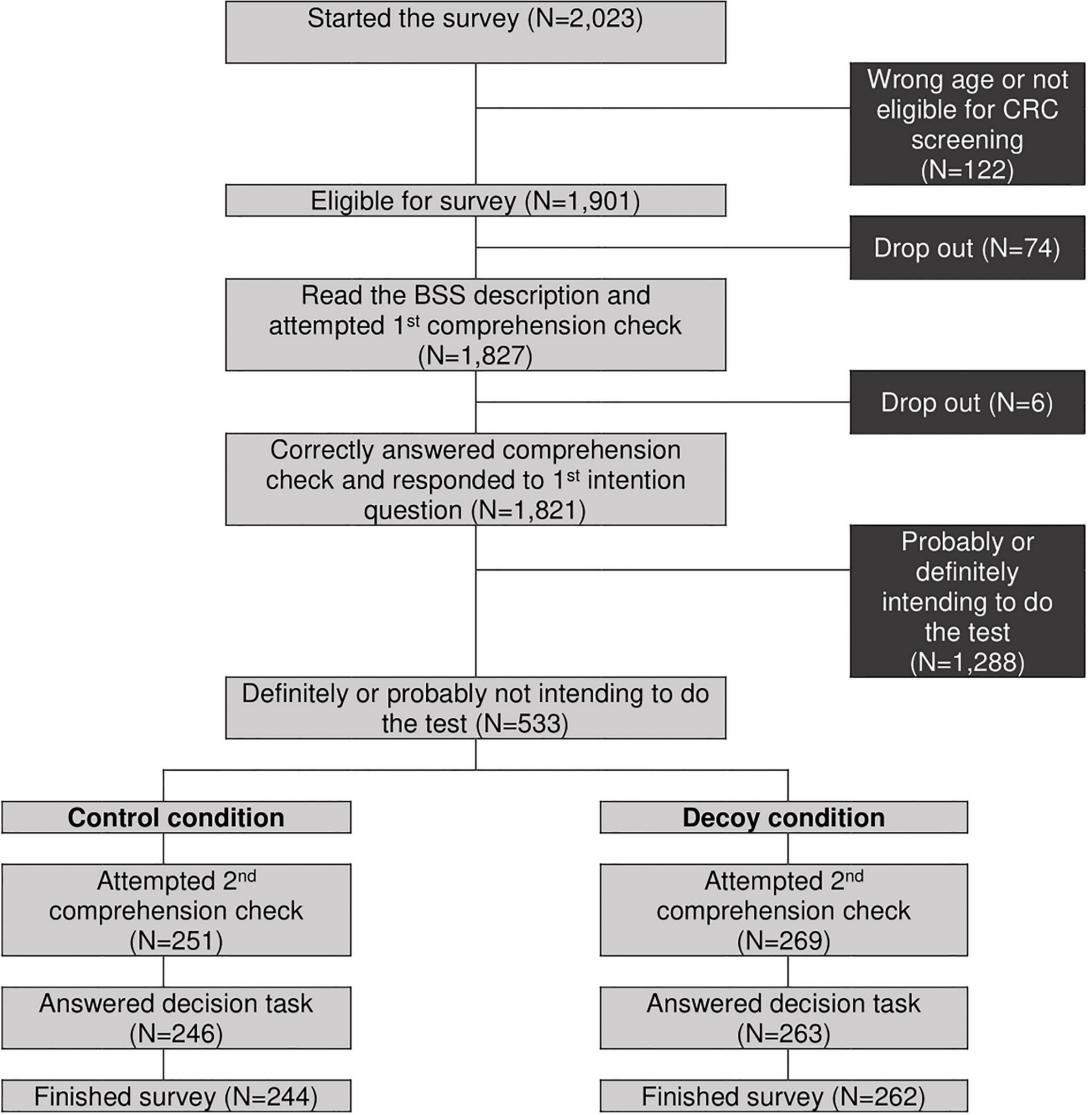
Flow though Study 1.

In Study 1, men and women aged 35–54 living in England were recruited from an English online panel (Survey Sampling International) and received a small financial incentive from the survey vendor, which was defined by the length of the questionnaire, for completing the survey (around 50 pence). Participants who were not filtered out or dropped out of the survey did not receive an incentive. At the start of the survey, participants were asked to give explicit consent for their data to be used and published as part of this research project, before they were presented with a short description of BSS and were asked to correctly answer a compulsory comprehension question regarding the screening procedure (Fig 2). Participants were then asked to indicate whether they would take up the offer if you were invited to have the test. Those who had indicated that they would probably or definitely have the test, were redirected to the study briefing and final survey page where they were thanked for their participation. Those who stated that they would definitely or probably not do the screening test were individually randomised, with equal probability, to one of two conditions [35].

In the control condition, participants were asked to choose between doing the screening test at the target hospital and no screening. In the decoy condition, the decoy option was added and participants could choose between doing the test at either of the two hospitals or no screening. Note that within each condition, eligible responders were randomised, with equal probability, to one of two versions of the scenario (travel vs. waiting time version).

#### Measures

Participants were asked to choose between no screening and attending screening at the offered hospital(s). To prevent order effects, we counterbalanced the order of the screening hospital(s) and the ‘no screening’ option and the order in which we presented the benefits and cost information about each option (see S2 Protocol for an example of the display). Furthermore, participants were asked to identify the correct travel or waiting time of the hospital(s) before being able to proceed to the choice question. Respondents in the decoy condition had an additional check that asked them to recall whether there were any differences between the decoy and target hospital in any other aspects, apart from the communicated attribute.

Perceived difficulty was collected on a fully labelled five-point Likert scale (‘Not at all’, ‘Slightly’, ‘Moderately’, ‘Very’ and ‘Extremely’) in response to the question ‘*How difficult was it for you to answer whether you would have the test at hospital X (or Hospital Y)*?’

A fully labelled five-point Likert scale (‘None’, ‘Little’, ‘Some’, ‘Considerate’, and ‘Great’) was used for the cognitive effort question ‘*How much effort did you put into deciding whether you would have the test at Hospital X (or Hospital Y)*?’

Both difficulty and effort questions were adapted and simplified from a 12-item subjective measurement of mental load and mental effort [36].

We assessed participants’ numeracy skills with the question ‘*Which of the following numbers represents the biggest risk of getting a disease*?’ with four possible answers ‘1/10’, ‘1/100’, ‘1/1000’, and ‘Don’t know’ [37].

We also measured cancer literacy through six questions adapted from a cancer health literacy test (CHLT-6) [38].

#### Sample size calculation

Sample sizes for both studies were calculated prior to data collection based on estimates obtained from pilot studies. Both studies were sufficiently powered to detect differences of at least 10% in participants choosing the target option between conditions, with a power of 80% and an alpha value of 0.05 [39].

### Results

#### Participants

In total, 2,023 individuals (aged 35–54) registered on a survey panel (Survey Sampling International) responded to an online survey invitation on BSS. 122 (6.0%) were excluded due to age or a self-reported previous diagnosis of colorectal cancer. Out of the 1,821 (90.0%) participants who read a description of BSS and passed the comprehension check, 533 (29.3%) indicated that they would either ‘definitely not’ (N = 110) or ‘probably not’ (N = 423) do the test. 1,288 (63.7%) with an intention to have the BSS (‘yes, probably’: N = 781; ‘yes, definitely’: N = 507) were eliminated at this point. 27 respondents (5.1%) did not finish the survey, leaving us with a final sample of 506 responders (244 in the control condition and 262 in the decoy condition) of whom 60.9% were female, 80.6% White-British, 53.2% married or cohabiting, 66.8% in paid employment and 57.9% had A-level or higher education. No statistical differences were found in sociodemographic characteristics, initial intention, numeracy skills or cancer literacy levels between the respondents in control and decoy conditions (see S2 Table).

#### Effect of decoy hospital on choice of screening hospitals

**Fig 3:**
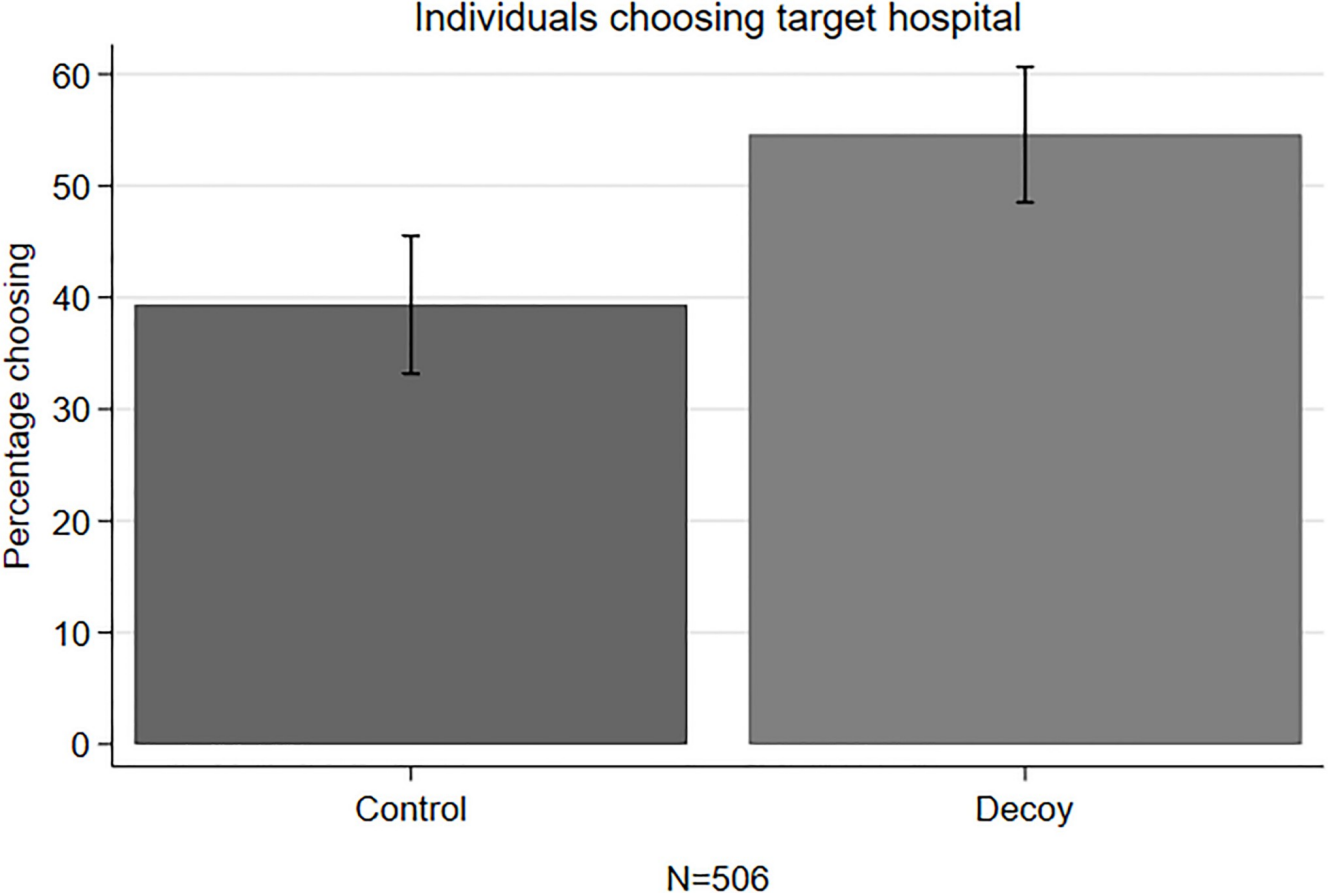
Mean percentage of choosing the target hospital.

Fig 3 shows that the proportion of participants who chose screening at the target hospital was significantly higher among those in the decoy as compared with those in the control condition (54.6% vs. 39.3%, χ^2^(1, N = 506) = 11.77, *p<*0.001). Note that the 15 responders (5.72%) who chose the decoy hospital in the decoy condition were classified as not wanting have the test at the target hospital.

Table 1 shows that this difference remained statistically significant after adjusting for covariates including initial intention, sociodemographic variables, cancer literacy score and numeracy skill (adjusted Odds Ratio (aOR) 1.89, 95% Confidence Interval (CI): 1.32–2.72, p < 0.01). Importantly, the difference was irrespective of whether the decoy was constructed around waiting or travel time, confirming that the attribute levels were well matched (see S1 Fig).

**Table 1:** Logistic regression models on choosing the target hospital.

	Unadjusted		Adjusted †	
	Odds ratio	95% CI	Odds ratio	95% CI
**Condition**				
Control	Ref.		Ref.	
Decoy	1.853	1.301–2.639**	1.890	1.315–2.716**
**Initial intention**				
Definitely not			Ref.	
Probably not			2.223	1.383–3.573**
*N*	506		506	
*R*^*2*^	0.031		0.081	

* *p*<0.05;

** *p*<0.01

^†^ Covariates included in the adjusted models are responder’s age, gender, marital status, ethnicity, education level, employment status, numeracy skill and cancer literacy score. Full model is included in the Supplementary file (see S3 Table).

#### Effect on choice difficulty and decisional effort

Ordered logistic regression models in Table 2 show that participants perceived making a decision as less difficult in the presence of the decoy (aOR 0.60, 95%CI: 0.43–0.84, p<0.01) and less effortful (aOR 0.48, 95%CI: 0.34–0.66, p<0.01).

**Table 2:** Ordered logistic regression models on perceived difficulty and cognitive effort.

	Perceived difficulty [1;5]				Cognitive effort [1;5]			
	Unadjusted		Adjusted †		Unadjusted		Adjusted †	
	Odds ratio	95% CI	Odds ratio	95% CI	Odds ratio	95% CI	Odds ratio	95% CI
**Condition**								
Control	Ref.		Ref.		Ref.		Ref.	
Decoy	0.587	0.422–0.818**	0.597	0.426–0.837**	0.497	0.360–0.686**	0.477	0.344–0.660**
**Initial intention**								
Definitely not			Ref.				Ref.	
Probably not			2.202	1.378–3.518**			2.564	1.668–3.941**
Cancer literacy (cont.)			0.708	0.612–0.818**			0.920	0.799–1.059
*N*	506		506		506		506	

* *p*<0.05;

** *p*<0.01

^†^ Covariates included in the adjusted models are responder’s age, gender, marital status, ethnicity, education level, paid employment and numeracy skills (See full models in S3 Table)

Results of the first experiment confirmed that adding a decoy increased the proportion of previous non-intenders choosing screening at the target hospital. These findings were an important proof of principle, that the lack of familiarity, and the complexity of the decision did not undermine this otherwise well-established decoy effect. However, Study 1 only manipulated one of two attributes at a time to characterise the hospital options and operationalise the decoy. The next step to better understand the mechanism behind this effect was to manipulate both attributes at the same time to create variations within decoy strength.

Furthermore, Study 2 addressed the concern that nudge type interventions subvert conscious deliberation and ‘lure’ people into making uninformed or misinformed choices. This view might indeed be further supported by our observation that respondents in the decoy group described their decision as easier and less burdensome.

## Study 2

The second study aimed to test whether dominance strength (i.e. the degree to which the decoy is dominated by the target) would moderate the decoy effect observed in Study 1. To vary dominance strength we manipulated travel and waiting time simultaneously to create a weak decoy (one of two attributes is undesirable) and a strong decoy (both attributes are undesirable). The second aim was to test whether the decoys would influence information seeking and knowledge about the harms and benefits of BSS.

### Method

#### Procedure

**Fig 4:**
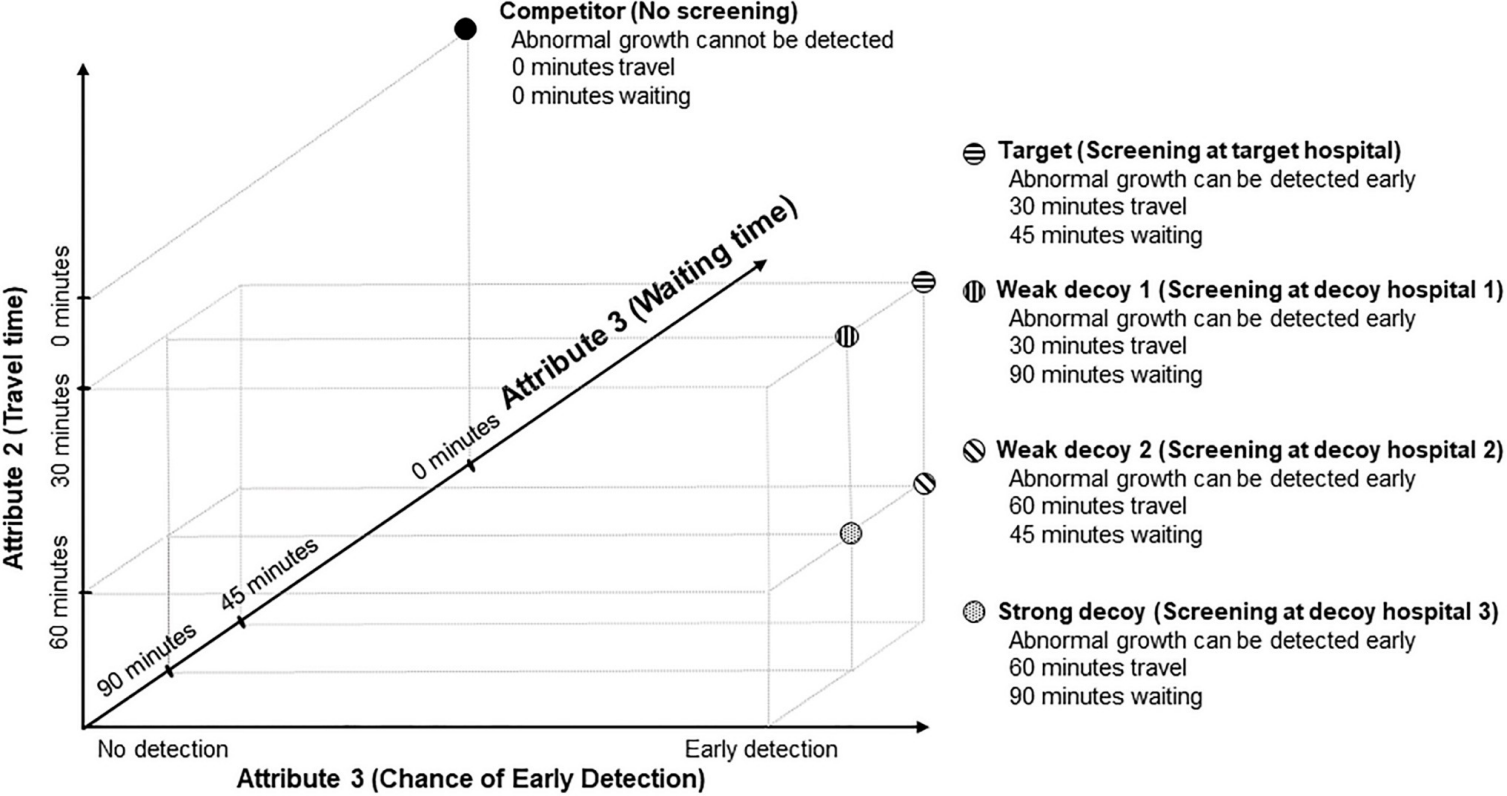
Implementation of the decoy effect in Study 2.

The procedure of the second study followed Study 1 in that after eligibility screening, responders were randomly allocated into three experimental conditions; control (screening at target hospital vs. no screening), weak decoy (target vs. weak decoy hospital vs. no screening) and strong decoy (target vs. strong decoy hospital vs. no screening) with equal probability. Fig 4 shows how the hospitals were defined: (1) the standard hospital required 30 minutes travel time and 45 minutes waiting time (target hospital), (2) the weak decoy hospital was inferior to the target hospital in either waiting time (decoy hospital 1) or travel time (decoy hospital 2), and (3) the strong decoy was inferior in both travel time and waiting time at the same time (decoy hospital 3). We again employed the same comprehension checks as in Study 1.

#### Measures

All outcome variables were the same as in Study 1 with the addition of a measure of ‘active interest’. Participants were asked whether they would like to ‘read’ or ‘skip’ more facts and figures about BSS. Those who indicated that they would like to skip it were sent to the end of the questionnaire, while those who wanted to read the information were presented additional information about BSS from the official NHS leaflet. Engagement with the information was measured with three comprehension questions.

### Results

#### Participants

**Fig 5:**
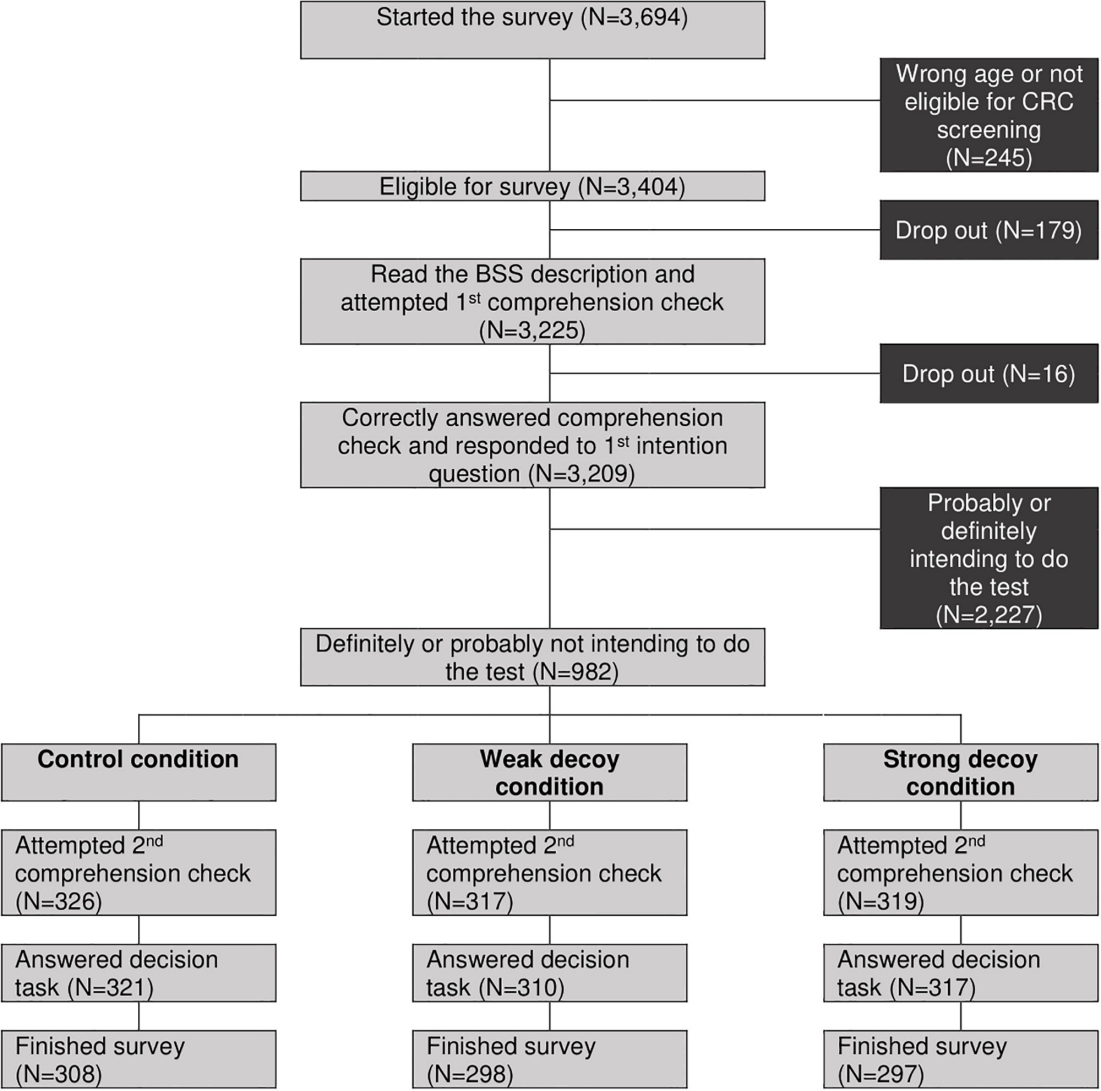
Flow though Study 2.

Fig 5 shows the study flow through the survey. While 982 (26.9%) of the initially invited 3,649 individuals were eligible based on age, no previous colorectal cancer diagnosis/bowel resection and no intention to participate in BSS (‘definitely not’: N = 236, 24.0%; ‘probably not’: N = 746, 76.0%), 903 (92.0%) finished the survey, (308 in the control condition, 298 in the weak decoy and 297 in the strong decoy condition). Sociodemographic findings were similar to Study 1 and variables were balanced between the three experimental conditions (see S4 Table). Most respondents were female (67.7%), White-British (83.9%), married or cohabiting (59.4%), in paid employed (68.8%) and had A-level or higher education (54.5%).

#### Effect of decoy hospital with various strength on choice of screening hospitals

**Fig 6:**
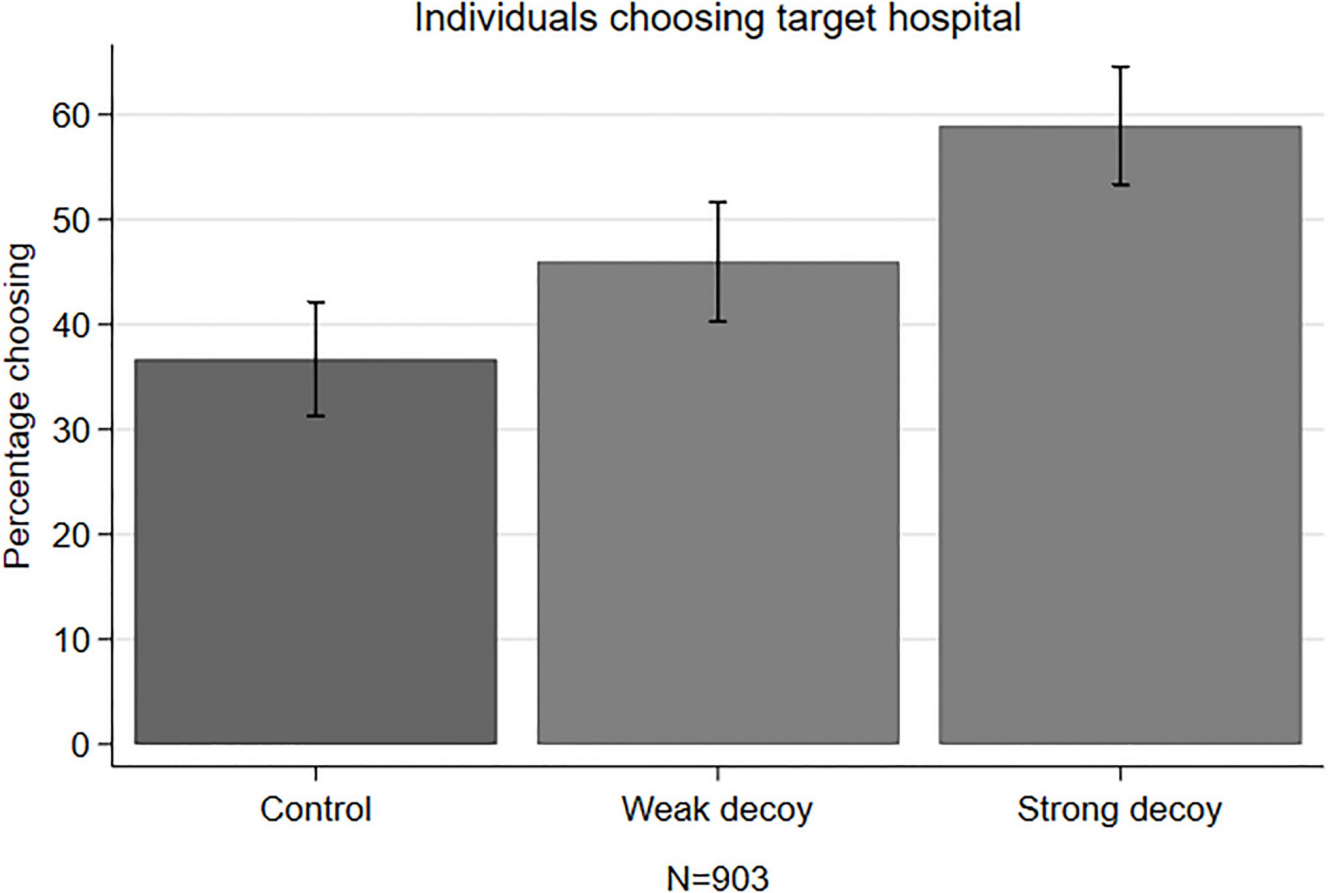
Mean percentage of choosing the target hospital.

Fig 6 shows that the proportion of respondents who chose screening at the target hospital was the highest among those in the strong decoy, followed by those in the weak decoy and those in the control condition (58.9% vs. 46.0% vs. 36.7%, χ^2^(2, N = 903) = 30.22, *p<*0.001). 33 responders (5.55%) chose the decoy hospital in one of the two decoy conditions and were classified as not wanting have the test at the target hospital (see S2 Fig).

The multivariate logistic regression reported in Table 3 confirms that the exposure to strong decoy hospital increased the probability of participants choosing to have BSS at the target hospital by around 2.7 times compared to control condition (aOR 2.66, 95%CI: 1.89–3.75, p<0.01), while the exposure to weak decoy increased the probability by 1.6 times (aOR 1.61, 95%CI: 1.15–2.25, p<0.01).

**Table 3:** Logistic regression models on hospital choice in Study 2.

	Unadjusted		Adjusted †	
	Odds ratio	95% CI	Odds ratio	95% CI
**Condition**				
Control	Ref.		Ref.	
Weak decoy	1.468	1.061–2.032*	1.608	1.148–2.254**
Strong decoy	2.475	1.784–3.434**	2.662	1.891–3.746**
**Initial intention**				
Definitely not			Ref.	
Probably not			2.744	1.939–3.884**
*N*	903		903	
*R*^*2*^	0.044		0.126	

* *p*<0.05;

** *p*<0.01

^†^ Covariates included in the adjusted models are responder’s age, gender, marital status, ethnicity, education level, employment status, numeracy skills and cancer literacy score. Full model is included in the Supplementary file (see S5 Table).

The strong decoy also had a significantly stronger effect on choosing the target hospital compared with the weak decoy (aOR 1.66, 95% CI: 1.18–2.32, p<0.01).

#### Effect on perceived difficulty and cognitive effort

Similarly to Study 1, Table 4 shows that responders in the decoy conditions found the choice scenarios less difficult than those in the control condition (strong decoy: aOR 0.40, 95%CI 0.29–0.55, p<0.01; weak decoy: aOR 0.35, 95%CI 0.25–0.48, p<0.01) and that they spent less cognitive effort in making the decision (strong decoy: aOR 0.57, 95%CI: 0.42–0.76, p<0.01; weak decoy: aOR 0.553, 95%CI: 0.41–0.74, p<0.01). There were, however, no statistically significant differences between the strong and weak decoy conditions.

**Table 4:** Ordered logistic regression models on perceived difficulty and cognitive effort in Study 2.

	Perceived difficulty [1;5]				Cognitive effort [1;5]			
	Unadjusted		Adjusted †		Unadjusted		Adjusted †	
	Odds ratio	95% CI	95% CI	Odds ratio	95% CI	95% CI	95% CI	Odds ratio
**Condition**								
Control	Ref.		Ref.		Ref.		Ref.	
Weak decoy	0.354	0.258–0.484**	0.346	0.250–0.478**	0.523	0.391–0.700**	0.553	0.413–0.742**
Strong decoy	0.417	0.305–0.569**	0.401	0.292–0.552**	0.554	0.415–0.741**	0.566	0.421–0.759**
**Initial intention**								
Definitely not			Ref.				Ref.	
Probably not			2.400	1.688–3.412**			2.097	1.556–2.825**
Cancer literacy (cont.)			0.724	0.654–0.801**			0.924	0.839–1.019
*N*	903		903		903		903	

* *p*<0.05;

** *p*<0.01

^†^ Covariates included in the adjusted models are responder’s age, gender, marital status, ethnicity, education level, paid employment and numeracy skills (See full models in S5 Table)

#### Effect on information seeking and engagement

**Fig 7:**
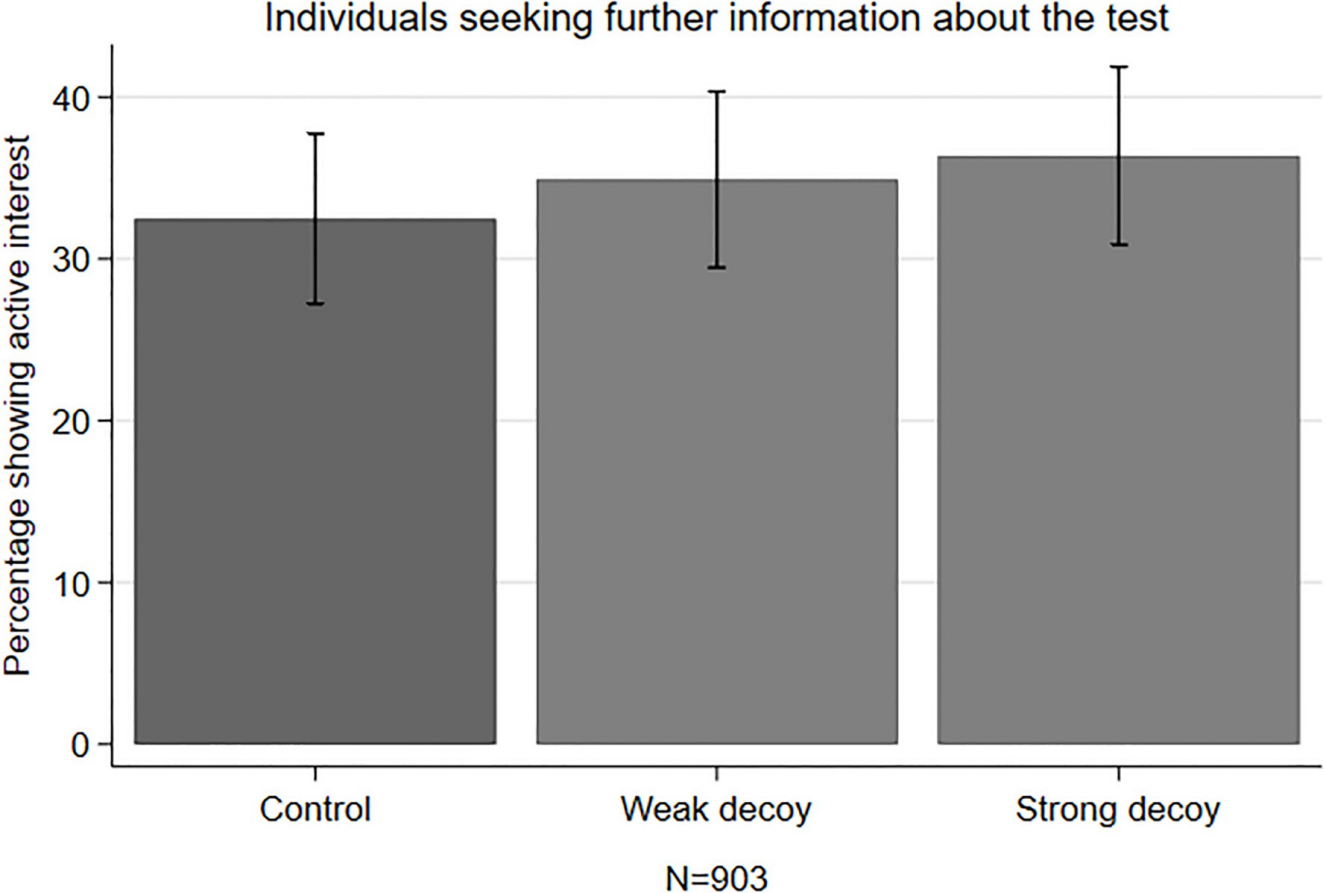
Mean percentage of seeking more information.

Fig 7 shows that the decoy did not influence interest in reading more information about the screening programme (see also S3 Fig). Independently from the experimental condition, around one third of the respondents stated that they wanted to read more (32.5–36.4%, χ^2^(2, N = 903) = 1.04, *p* = 0.595). A Kruskal-Wallis test did not reveal any differences in BSS knowledge across the conditions (χ^2^ = 2.59, p = 0.274, df = 2). Most participants who read the additional information got around 2 out of 3 comprehension questions right.

A comparison of those who selected the target in one of the three conditions, reveals that a higher proportion of intenders in the two decoy conditions wanted to read additional information compared with those selecting the target in the control condition (40.9% in the weak decoy vs. 44.6% in the strong decoy vs. 28.3% in the control condition, χ^2^(2, N = 425) = 7.90, *p* = 0.019). Finally, while not being statistically significant, intenders in the decoy condition had higher knowledge scores compared with those in the control condition (χ^2^ = 5.23, p = 0.073, df = 2). Most intenders in the decoy conditions answered 2 out of 3 comprehension questions correctly, while most intenders in the control condition only answered 1 correctly.

Study 2 demonstrated that a positive relationship between the decoy effect and the domination strength on interest in doing the test at the target hospital. The stronger the target hospital dominated the decoy, the more likely individuals were to choose the target. Importantly, despite making the decision easier and less burdensome, being in the decoy condition did not adversely affect information seeking or BSS knowledge.

## General discussion

This is the first study that tested the decoy effect in cancer screening. In two experimental online surveys, we showed that individual screening intentions can be increased by adding a decoy screening alternative to the choice set. Furthermore, we found that the decoy effect is influenced by the degree to which the decoy option was dominated by the target option. This shows that introducing carefully designed screening alternatives can not only increase the appeal of the target but also avoid choice overload [40].

Our study samples consisted of participants who were initially not intending to attend screening. This is important as Huber and colleagues suggested that initial preferences could influence the decoy effect [3]. Having already a strong preference for non-screening could mean that individuals may fail to perceive the dominance relationship between decoy and target. In our studies, initial strong disinclination (‘definitely not’ having the test) independently reduced the likelihood of respondents choosing target, however, one of the strengths of our study was that the proportion of participants with different initial intentions was well-balanced between the experimental arms in both studies. Moreover, the decoy effect remained significant after adjusting for all variables including initial intention.

Importantly, as our decoys did not negatively influence information seeking behaviour in the second study, this particular nudge technique is unlikely to undermine people’s ability to make an informed choice about participating in cancer screening. On the contrary, individuals in the decoy conditions who selected the target were more likely to read the additional information than those selecting the target in the control condition.

The study had several limitations. Following previous research on the decoy effect, we used an approach that made all decision options explicit. Thus, in our experiment non-screening was a salient option. While one could argue that hiding the non-screening option would change the appearance of the decision setting, a recent study confirmed the decoy effect when the competitor (not engaging in the behaviour) was hidden [14].

Perhaps the most important limitation at this stage was the lack of behavioural validation.

We measured screening intentions in a hypothetical scenario, and not real behaviour. Literature on the intention-behaviour gap suggests that although individuals may develop an intention to get screened, they might not necessarily take any action to get screened [41]. In this study, around 70% stated initially that would do the test, while in reality only around 43% do it [20]. Furthermore, our study samples consisted of men and women aged 35 to 54 who were not screening eligible at the time of the survey and not representative for the overall population, which limits external validity of our study.

However, in line with the recently advocated experimental medicine approach [42] proof of principle studies like this are vitally important because they allow us to test the effect of potential strategies under well-controlled conditions, while field experiments can fail to show effects for reasons that are more to do with translational issues (e.g. how messages can be delivered and processed in real world contexts) than the efficacy of the specific technique. Now that we have shown the decoys can work in an online setting, the next step would be to test it under more ecologically valid conditions.

Finally, our findings have significant potential implications for not only cancer screening programmes, but decision making about medical treatments in general. In a practical sense, while our study shows that decoys could facilitate the screening decision and increase participation rates, future studies should investigate how decoys could be used to influence other screening programmes and elective surgery treatments (e.g. offering alternative appointments with a non-specialist to boost the attractiveness of the specialist). Furthermore, in line with ethical concerns about nudging people into choosing medical treatment options, the decoy effect could be used to nudge people into engaging with information about the medical treatment to foster informed decision-making (i.e. offer alternative information formats). For now, the observation that the presence of a decoy does not undermine informed choice is reassuring. Policy makers that wish to use behavioural interventions based on the decoy effect should be aware that they may result in unexpected choices which could have logistical and ethical implications (i.e. the decoy must be carefully designed so that it does not increase the attractiveness of the socially undesirable alternative).

## Supporting information

S1 Protocol Sample questions used in the preliminary studies. (DOCX)

S2 Protocol Examples of the choice sets presented to respondents in Study 1 and 2. (DOCX)

S1 Table Descriptive statistics of the study populations in the preliminary studies. (DOCX)

S2 Table Descriptive statistics of the study population in Study 1. (DOCX)

S3 Table Multivariate regression models for Study 1. (DOCX)

S4 Table Descriptive statistics of the study population in Study 2. (DOCX)

S5 Table Multivariate regression models for Study 2. (DOCX)

S1 Fig Mean percentage of choosing the target hospital in Study 1. (DOCX)

S2 Fig Mean percentage of choosing the target hospital in Study 2. (DOCX)

S3 Fig Mean percentage of seeking more information in Study 2. (DOCX)
