# Correction: Testing the decoy effect to increase interest in colorectal cancer screening

**DOI:** 10.1371/journal.pone.0219811

**Published:** 2019-07-10

**Authors:** Sandro Tiziano Stoffel, Jiahong Yang, Ivo Vlaev, Christian von Wagner

After publication of this article [1], the paper cited in Reference 14 was retracted by *Psychological Science*. In the second paragraph of the Introduction, Reference 14 is cited as evidence supporting the decoy effect in promoting health related behaviors. In the fourth paragraph of the General discussion section, Reference 14 is cited to show that a decoy effect can occur in the absence of a salient competitor.

In light of the retraction, the authors of [1] would like to clarify that Reference 14 should no longer be considered as evidence in support of the decoy effect. Furthermore, the authors maintain that the retraction of the cited work does not affect the results or overall scientific understanding of the study.

## References

[1] Stoffel ST, Yang J, Vlaev I, von Wagner C (2019) Testing the decoy effect to increase interest in colorectal cancer screening. PLoS ONE 14(3): e0213668 10.1371/journal.pone.0213668 30913209 PMC6435152

