# Supplementary material for: Testing the decoy effect to increase interest in colorectal cancer screening
Source: PLoS One. 2019 Mar 26;14(3):e0213668. doi: 10.1371/journal.pone.0213668 (PMC6435152; doi:10.1371/journal.pone.0213668)
Supplement: S1 Protocol — (DOCX) [file pone.0213668.s001.docx]

# S1 Protocol: Sample questions used in the preliminary studies

Imagine that you have received an invitation letter from your local screening centre to do the test at a hospital. There are two hospitals available who would be available for BSS. Both hospitals only differ in their average waiting time at site and travel time. Thus, apart from the waiting and travel time, there are no further differences between the two hospitals such as quality of service, parking space or costs.

In your case, you have been offered to do the BSS either at Hospital X or at Hospital Y. Please indicate for each of the following comparisons which of the two hospitals you prefer.

| Task | Hospital X | Hospital Y |
| --- | --- | --- |
| 1 | **30** minutes travel time and  **0** minutes waiting time on site | **0** minutes travel time and  **0** minutes waiting time on site |
| 2 | **30** minutes travel time and  **0** minutes waiting time on site | **0** minutes travel time and  **5** minutes waiting time on site |
| 3 | **30** minutes travel time and  **0** minutes waiting time on site | **0** minutes travel time and  **10** minutes waiting time on site |
| 4 | **30** minutes travel time and  **0** minutes waiting time on site | **0** minutes travel time and  **15** minutes waiting time on site |
| 5 | **30** minutes travel time and  **0** minutes waiting time on site | **0** minutes travel time and  **20** minutes waiting time on site |
| 6 | **30** minutes travel time and  **0** minutes waiting time on site | **0** minutes travel time and  **25** minutes waiting time on site |
| 7 | **30** minutes travel time and  **0** minutes waiting time on site | **0** minutes travel time and  **30** minutes waiting time on site |
| 8 | **30** minutes travel time and  **0** minutes waiting time on site | **0** minutes travel time and  **35** minutes waiting time on site |
| 9 | **30** minutes travel time and  **0** minutes waiting time on site | **0** minutes travel time and  **40** minutes waiting time on site |
| 10 | **30** minutes travel time and  **0** minutes waiting time on site | **0** minutes travel time and  **45** minutes of waiting time on site |
| 11 | **30** minutes travel time and  **0** minutes waiting time on site | **0** minutes travel time and  **50** minutes of waiting time on site |
| 12 | **30** minutes travel time and  **0** minutes waiting time on site | **0** minutes travel time  **55** minutes of waiting time on site |
| 13 | **30** minutes travel time and  **0** minutes waiting time on site | **0** minutes travel time and  **60** minutes or more of waiting time on site |
