# Supplementary material for: Testing the decoy effect to increase interest in colorectal cancer screening
Source: PLoS One. 2019 Mar 26;14(3):e0213668. doi: 10.1371/journal.pone.0213668 (PMC6435152; doi:10.1371/journal.pone.0213668)
Supplement: S2 Protocol — (DOCX) [file pone.0213668.s002.docx]

# S3 Protocol: Examples of the choice sets presented to respondents in Study 1 (on top) and 2 (on bottom)

*
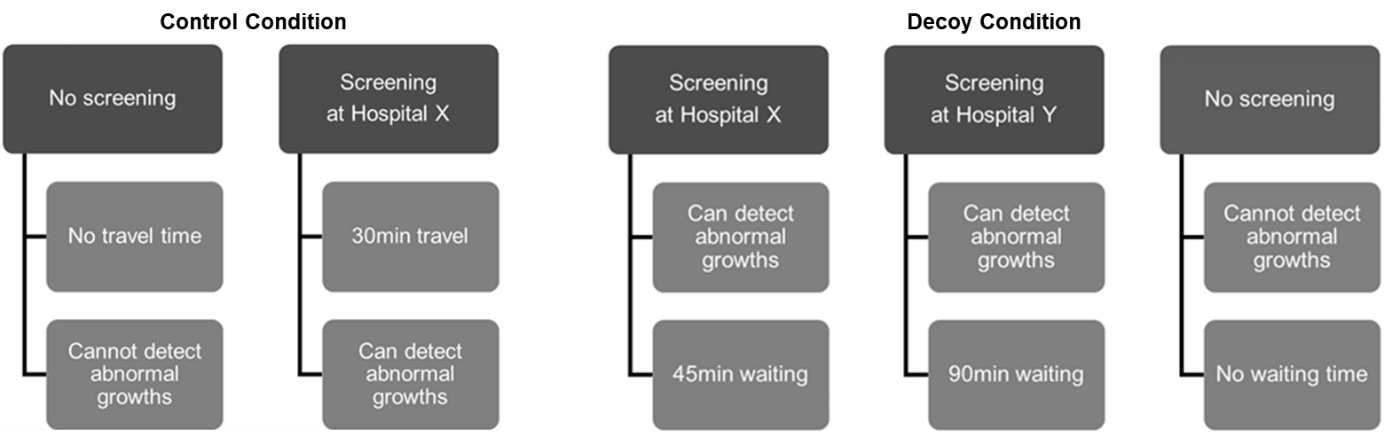
*

*
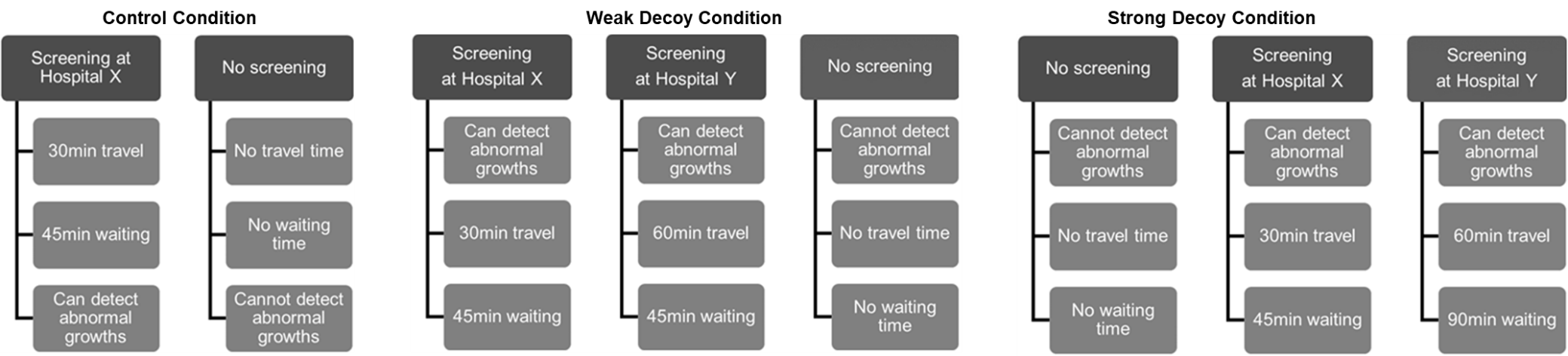
*

*Examples of the choice sets presented to participants in Study 1 and 2. In control condition, participants would only be exposed to choice options of “no screening” (Competitor) and “screening at Hospital X” (Target). In the Decoy condition, participants had an additional choice option of “screening at Hospital Y” which was inferior in either travel or waiting time. The order of presenting each option (i.e. Target, Competitor or Decoy) and each attribute (i.e. chance of detection, waiting time and travel time) was randomised to prevent order effect.*
