# Supplementary material for: Testing the decoy effect to increase interest in colorectal cancer screening
Source: PLoS One. 2019 Mar 26;14(3):e0213668. doi: 10.1371/journal.pone.0213668 (PMC6435152; doi:10.1371/journal.pone.0213668)
Supplement: S1 Table — (DOCX) [file pone.0213668.s003.docx]

# S1 Table: Descriptive statistics of the study populations in the preliminary studies (N=118)

|  |  | Round 1  (N=60) | | Round 2  (N=58) | | Overall  (N=118) | | p-value* |
| --- | --- | --- | --- | --- | --- | --- | --- | --- |
| **Age** | |  |  |  |  |  |  |  |
|  | 35-44 | 29 | (48.3%) | 27 | (46.6%) | 56 | (47.5%) | 0.993 |
|  | 45-54 | 31 | (51.7%) | 31 | (53.5%) | 62 | (52.5%) |  |
| **Gender** | |  |  |  |  |  |  |  |
|  | Male | 37 | (61.7%) | 32 | (55.2%) | 69 | (58.5%) | 0.597 |
|  | Female | 23 | (38.3%) | 26 | (44.8%) | 49 | (41.5%) |  |
| **Living status** | |  |  |  |  |  |  |  |
|  | Single/div./wid.✝ | 29 | (48.3%) | 29 | (50.0%) | 58 | (49.2%) | 0.856 |
|  | Married/cohabiting | 31 | (51.7%) | 29 | (50.0%) | 60 | (50.8%) |  |
| **Ethnicity** | |  |  |  |  |  |  |  |
|  | White British | 48 | (80.0%) | 48 | (82.8%) | 96 | (81.4%) | 0.882 |
|  | Other | 12 | (20.0%) | 10 | (17.2%) | 22 | (18.6%) |  |
| **Education** | |  |  |  |  |  |  |  |
|  | No A levels | 24 | (40.0%) | 24 | (41.4%) | 48 | (40.7%) | 0.879 |
|  | A levels or higher | 36 | (60.0%) | 34 | (58.6%) | 70 | (59.3%) |  |
| **Paid employment** | |  |  |  |  |  |  |  |
|  | No | 28 | (46.7%) | 19 | (32.8%) | 47 | (39.8%) | 0.176 |
|  | Yes | 32 | (53.3%) | 39 | (67.2%) | 71 | (60.2%) |  |
| **Intentions before exposure** | | |  |  |  |  |  |  |
|  | Definitely not | 12 | (20.0%) | 19 | (32.8%) | 31 | (26.3%) | 0.172 |
|  | Probably not | 48 | (80.0%) | 39 | (67.2%) | 87 | (79.6%) |  |

*p-value refers to Chi-Square test of independence if not stated differently.

‡p-value refers to two sample t test.

✝Single, divorced or widowed
