# Supplementary material for: Testing the decoy effect to increase interest in colorectal cancer screening
Source: PLoS One. 2019 Mar 26;14(3):e0213668. doi: 10.1371/journal.pone.0213668 (PMC6435152; doi:10.1371/journal.pone.0213668)
Supplement: S2 Table — (DOCX) [file pone.0213668.s004.docx]

# S2 Table: Descriptive statistics of the study population in Study 1 (N=506)

|  |  | Control  (N=244) | | Decoy  (N=262) | | Overall  (N=506) | | p-value* |
| --- | --- | --- | --- | --- | --- | --- | --- | --- |
| **Age** | |  |  |  |  |  |  |  |
|  | 35-44 | 128 | (52.5%) | 141 | (53.8%) | 269 | (53.2%) | 0.760 |
|  | 45-54 | 116 | (47.5%) | 121 | (46.2%) | 237 | (46.8%) |  |
| **Gender** | |  |  |  |  |  |  |  |
|  | Male | 101 | (41.4%) | 97 | (37.0%) | 198 | (39.1%) | 0.314 |
|  | Female | 143 | (58.6%) | 165 | (63.0%) | 308 | (60.9%) |  |
| **Living status** | |  |  |  |  |  |  |  |
|  | Single/div./wid.✝ | 168 | (68.9%) | 172 | (65.6%) | 340 | (67.2%) | 0.381 |
|  | Married/cohabiting | 76 | (31.1%) | 90 | (34.4%) | 166 | (32.8%) |  |
| **Ethnicity** | |  |  |  |  |  |  |  |
|  | White British | 197 | (80.7%) | 211 | (80.5%) | 408 | (80.6%) | 0.443 |
|  | Other | 47 | (19.3%) | 51 | (19.5%) | 98 | (19.4%) |  |
| **Education** | |  |  |  |  |  |  |  |
|  | No A levels | 106 | (43.4%) | 107 | (40.8%) | 213 | (42.1%) | 0.553 |
|  | A levels or higher | 138 | (56.6%) | 155 | (59.2%) | 293 | (57.9%) |  |
| **Paid employment** | |  |  |  |  |  |  |  |
|  | No | 77 | (31.6%) | 91 | (34.7%) | 168 | (33.2%) | 0.449 |
|  | Yes | 167 | (68.4%) | 171 | (65.3%) | 338 | (66.8%) |  |
| **Numeracy question** | |  |  |  |  |  |  |  |
|  | Wrong | 129 | (52.9%) | 136 | (51.9%) | 265 | (52.4%) | 0.829 |
|  | Correct | 115 | (47.1%) | 126 | (48.1%) | 241 | (47.6%) |  |
| **Cancer literacy (Score 0-6)** | | | |  |  |  |  |  |
|  | Mean and SD | 4.90 | 1.31 | 5.02 | 1.16 | 4.96 | 1.23 | 0.284‡ |
| **Intentions before exposure** | | |  |  |  |  |  |  |
|  | Definitely not | 55 | (22.5%) | 48 | (18.3%) | 103 | (20.4%) | 0.239 |
|  | Probably not | 189 | (77.5%) | 214 | (81.7%) | 403 | (79.6%) |  |

*p-value refers to Chi-Square test of independence if not stated differently.

‡p-value refers to two sample t test.

✝Single, divorced or widowed
