# Supplementary material for: Testing the decoy effect to increase interest in colorectal cancer screening
Source: PLoS One. 2019 Mar 26;14(3):e0213668. doi: 10.1371/journal.pone.0213668 (PMC6435152; doi:10.1371/journal.pone.0213668)
Supplement: S3 Table — (DOCX) [file pone.0213668.s005.docx]

# S3 Table: Multivariate regression models for Study 1

|  | Choosing target hospital [0;1] | | Perceived difficulty [1;5] | | Cognitive effort [1;5] | |
| --- | --- | --- | --- | --- | --- | --- |
|  | Odds ratio | 95% CI | Odds ratio | 95% CI | Odds ratio | 95% CI |
| **Condition** |  |  |  |  |  |  |
| Control | Ref. |  | Ref. |  | Ref. |  |
| Decoy | 1.880 | 1.309 - 2.701** | 0.597 | 0.426 - 0.837** | 0.475 | 0.343 - 0.658** |
| **Initial intentions** | |  |  |  |  |  |
| Definitely not | Ref. |  | Ref. |  | Ref. |  |
| Probably not | 2.222 | 1.384 - 3.569** | 2.202 | 1.378 - 3.518** | 2.555 | 1.662 - 3.930** |
| **Age** |  |  |  |  |  |  |
| 35-44 years | Ref. |  | Ref. |  | Ref. |  |
| 45-54 years | 0.787 | 0.543 - 1.140 | 0.804 | 0.569 - 1.137 | 0.925 | 0.668 - 1.282 |
| **Gender** |  |  |  |  |  |  |
| Male | Ref. |  | Ref. |  | Ref. |  |
| Female | 0.981 | 0.671 - 1.436 | 1.005 | 0.705 - 1.432 | 0.967 | 0.692 - 1.350 |
| **Marital status** | |  |  |  |  |  |
| Single/div./wid. | Ref. |  | Ref. |  | Ref. |  |
| Married/cohab. | 1.058 | 0.732 - 1.529 | 1.009 | 0.717 - 1.420 | 0.780 | 0.563 - 1.081 |
| **Ethnicity** |  |  |  |  |  |  |
| White British | Ref. |  | Ref. |  | Ref. |  |
| Other | 1.230 | 0.766 - 1.976 | 0.974 | 0.624 - 1.519 | 0.935 | 0.617 - 1.418 |
| **A-levels** |  |  |  |  |  |  |
| No | Ref. |  | Ref. |  | Ref. |  |
| Yes | 0.949 | 0.636 - 1.414 | 1.028 | 0.708 - 1.493 | 0.957 | 0.672 - 1.364 |
| **Paid employment** | |  |  |  |  |  |
| No | Ref. |  | Ref. |  | Ref. |  |
| Yes | 1.132 | 0.764 - 1.678 | 1.061 | 0.735 - 1.531 | 1.037 | 0.734 - 1.465 |
| **Numeracy question** | |  |  |  |  |  |
| Wrong | Ref. |  | Ref. |  | Ref. |  |
| Correct | 0.944 | 0.649 - 1.372 | 0.813 | 0.572 - 1.155 | 0.934 | 0.673 - 1.298 |
| **Cancer literacy** | |  |  |  |  |  |
| Score (0-6) | 0.857 | 0.729 - 1.008 | 0.708 | 0.612 - 0.818** | 0.918 | 0.798 - 1.057 |
| N | 506 |  | 506 |  | 506 |  |

* *p*<0.05; ** *p*<0.01
