# Supplementary material for: Testing the decoy effect to increase interest in colorectal cancer screening
Source: PLoS One. 2019 Mar 26;14(3):e0213668. doi: 10.1371/journal.pone.0213668 (PMC6435152; doi:10.1371/journal.pone.0213668)
Supplement: S4 Table — (DOCX) [file pone.0213668.s006.docx]

# S4 Table: Descriptive statistics of the study population in Study 2 (N=903)

|  |  | Control  (N=308) | | Weak decoy  (N=298) | | Strong decoy  (N=297) | | Overall  (N=903) | | p-value* |
| --- | --- | --- | --- | --- | --- | --- | --- | --- | --- | --- |
| **Age** | |  |  |  |  |  |  |  |  |  |
|  | 35-44 | 167 | (54.2%) | 156 | (52.3%) | 165 | (55.6%) | 488 | (54.0%) | 0.733 |
|  | 45-54 | 141 | (45.8%) | 142 | (47.7%) | 132 | (44.4%) | 415 | (46.0%) |  |
| **Gender** | |  |  |  |  |  |  |  |  |  |
|  | Male | 99 | (32.1%) | 83 | (27.9%) | 110 | (37.0%) | 292 | (32.3%) | 0.057 |
|  | Female | 209 | (67.9%) | 215 | (72.1%) | 187 | (63.0%) | 611 | (67.7%) |  |
| **Living status** | |  |  |  |  |  |  |  |  |  |
|  | Single/div./wid.✝ | 135 | (43.8%) | 115 | (38.6%) | 117 | (39.4%) | 367 | (40.6%) | 0.366 |
|  | Married/cohabiting | 173 | (56.2%) | 183 | (61.4%) | 180 | (60.6%) | 536 | (59.4%) |  |
| **Ethnicity** | |  |  |  |  |  |  |  |  |  |
|  | White British | 264 | (85.7%) | 241 | (80.9%) | 253 | (85.2%) | 758 | (83.9%) | 0.208 |
|  | Other | 44 | (14.3%) | 57 | (19.1%) | 44 | (14.8%) | 145 | (16.1%) |  |
| **Education** | |  |  |  |  |  |  |  |  |  |
|  | No A levels | 136 | (44.2%) | 129 | (43.3%) | 146 | (49.2%) | 411 | (45.5%) | 0.299 |
|  | A levels or higher | 172 | (55.8%) | 169 | (56.7%) | 151 | (50.8%) | 492 | (54.5%) |  |
| **Paid employment** | | | |  |  |  |  |  |  |  |
|  | No | 97 | (31.5%) | 102 | (34.2%) | 83 | (27.9%) | 282 | (31.2%) | 0.253 |
|  | Yes | 211 | (68.5%) | 196 | (65.8%) | 214 | (72.1%) | 621 | (68.8%) |  |
| **Numeracy question** | | | |  |  |  |  |  |  |  |
|  | Wrong | 174 | (56.5%) | 175 | (58.7%) | 164 | (55.2%) | 513 | (56.8%) | 0.682 |
|  | Correct | 134 | (43.5%) | 123 | (41.3%) | 133 | (44.8%) | 390 | (43.2%) |  |
| **Cancer literacy (Score 0-6)** | | | |  |  |  |  |  |  |  |
|  | Mean and SD | 4.89 | 1.32 | 4.91 | 1.35 | 4.94 | 1.25 | 4.91 | 1.31 | 0.895‡ |
| **Intentions before exposure** | | | |  |  |  |  |  |  |  |
|  | Definitely not | 63 | (20.5%) | 76 | (25.5%) | 72 | (24.2%) | 211 | (23.4%) | 0.682 |
|  | Probably not | 245 | (79.5%) | 222 | (74.5%) | 225 | (75.8%) | 692 | (76.6%) |  |

*p-value refers to Chi-Square test of independence if not stated differently.

‡p-value refers to a one-way ANOVA.

✝Single, divorced or widowed.
