# Supplementary material for: Testing the decoy effect to increase interest in colorectal cancer screening
Source: PLoS One. 2019 Mar 26;14(3):e0213668. doi: 10.1371/journal.pone.0213668 (PMC6435152; doi:10.1371/journal.pone.0213668)
Supplement: S5 Table — (DOCX) [file pone.0213668.s007.docx]

# S5 Table: Multivariate regression models for Study 2

|  | Choosing target hospital [0;1] | | Perceived difficulty [1;5] | | Cognitive effort [1;5] | |
| --- | --- | --- | --- | --- | --- | --- |
|  | Odds ratio | 95% CI | Odds ratio | 95% CI | Odds ratio | 95% CI |
| **Condition** |  |  |  |  |  |  |
| Control | Ref. |  | Ref. |  | Ref. |  |
| Weak decoy | 1.608 | 1.148 - 2.254** | 0.346 | 0.250 - 0.478** | 0.553 | 0.413 - 0.742** |
| Strong decoy | 2.662 | 1.891 - 3.746** | 0.401 | 0.292 - 0.552** | 0.566 | 0.421 - 0.759** |
| **Initial intentions** | |  |  |  |  |  |
| Definitely not | Ref. |  | Ref. |  | Ref. |  |
| Probably not | 2.744 | 1.939 - 3.884** | 2.400 | 1.688 - 3.412** | 2.097 | 1.556 - 2.825** |
| **Age** |  |  |  |  |  |  |
| 35-44 years | Ref. |  | Ref. |  | Ref. |  |
| 45-54 years | 0.711 | 0.533 - 0.947* | 0.968 | 0.736 - 1.272 | 1.200 | 0.937 - 1.537 |
| **Gender** |  |  |  |  |  |  |
| Male | Ref. |  | Ref. |  | Ref. |  |
| Female | 1.030 | 0.761 - 1.395 | 0.769 | 0.580 - 1.018 | 1.005 | 0.777 - 1.299 |
| **Marital status** | |  |  |  |  |  |
| Single/div./wid. | Ref. |  | Ref. |  | Ref. |  |
| Married/cohab. | 1.086 | 0.819 - 1.440 | 0.958 | 0.732 - 1.253 | 1.006 | 0.789 - 1.283 |
| **Ethnicity** |  |  |  |  |  |  |
| White British | Ref. |  | Ref. |  | Ref. |  |
| Other | 1.003 | 0.682 - 1.474 | 1.114 | 0.776 - 1.599 | 0.718 | 0.514 - 1.005 |
| **A-levels** |  |  |  |  |  |  |
| No | Ref. |  | Ref. |  | Ref. |  |
| Yes | 1.050 | 0.784 - 1.408 | 1.104 | 0.835 - 1.460 | 1.062 | 0.824 - 1.368 |
| **Paid employment** | |  |  |  |  |  |
| No | Ref. |  | Ref. |  | Ref. |  |
| Yes | 1.600 | 1.175 - 2.179** | 1.054 | 0.784 - 1.418 | 1.270 | 0.971 - 1.662 |
| **Numeracy question** | |  |  |  |  |  |
| Wrong | Ref. |  | Ref. |  | Ref. |  |
| Correct | 1.160 | 0.873 - 1.543 | 0.878 | 0.666 - 1.156 | 0.981 | 0.767 - 1.255 |
| **Cancer literacy** | |  |  |  |  |  |
| Score (0-6) | 1.062 | 0.949 - 1.189 | 0.724 | 0.654 - 0.801** | 0.924 | 0.839 - 1.019 |
| N | 903 |  | 903 |  | 903 |  |

* *p*<0.05; ** *p*<0.01
