# Supplementary material for: Testing the decoy effect to increase interest in colorectal cancer screening
Source: PLoS One. 2019 Mar 26;14(3):e0213668. doi: 10.1371/journal.pone.0213668 (PMC6435152; doi:10.1371/journal.pone.0213668)
Supplement: S1 Fig — (DOCX) [file pone.0213668.s008.docx]

# S1 Figure: Mean percentage of choosing the target hospital with standard deviation error bars in Study 1

|  | | | | | | | | | | | |
| --- | --- | --- | --- | --- | --- | --- | --- | --- | --- | --- | --- |
|  | Control (Wait)  (N=119) | | Control (Travel)  (N=125) | | Decoy (Wait)  (N=105) | | Decoy (Travel)  (N=157) | | Overall  (N=506) | | p-value |
| Choosing target hospital | 72 | (60.5%) | 76 | (60.8%) | 48 | (45.7%) | 71 | (45.2%) | 239 | (47.2%) | 0.008 |
| Not wanting to do the test ✝ | 47 | (39.5%) | 49 | (39.2%) | 57 | (54.3%) | 86 | (54.8%) | 267 | (52.8%) |  |

*p-value refers to Chi-Square test of independence.

✝Also includes those 15 responders who chose one of the decoy hospitals in the decoy conditions. Specifically, 6 responders (5.7%) chose the decoy hospital in the Decoy (Wait) condition and 9 (5.7%) in the Decoy (Travel) condition.

There are no statistically significant differences between the two control conditions (39.2% vs. 39.5%, χ^2^(2, N=244) =0.002, p=0.962) and the two decoy conditions (54.3% vs. 54.8%, χ^2^(2, N=262) =0.006, p=0.938).
