# Supplementary material for: Testing the decoy effect to increase interest in colorectal cancer screening
Source: PLoS One. 2019 Mar 26;14(3):e0213668. doi: 10.1371/journal.pone.0213668 (PMC6435152; doi:10.1371/journal.pone.0213668)
Supplement: S2 Fig — (DOCX) [file pone.0213668.s009.docx]

# S2 Figure: Mean percentage of choosing the target hospital with standard deviation error bars in Study 2

|  | | | | | | | | | | | |
| --- | --- | --- | --- | --- | --- | --- | --- | --- | --- | --- | --- |
|  | Control  (N=308) | | Weak decoy (Travel)  (N=160) | | Weak decoy (Wait)  (N=138) | | Strong decoy  (N=297) | | Overall  (N=903) | | p-value |
| Choosing target hospital | 195 | (63.3%) | 81 | (50.6%) | 80 | (58.0%) | 122 | (41.1%) | 478 | (52.9%) | <0.001 |
| Not wanting to do the test ✝ | 113 | (36.7%) | 79 | (49.4%) | 58 | (42.0%) | 175 | (58.9%) | 425 | (47.1%) |  |

*p-value refers to Chi-Square test of independence.

✝Also includes those 33 responders who chose one of the decoy hospitals in the decoy conditions. Specifically, 8 responders (5.0%) chose the decoy hospital in the Weak (Travel) condition, 6 (4.4%) chose it in the Weak (Wait) condition and 19 (6.4%) in the Strong Decoy condition.

There are no statistically significant differences between the two weak decoy conditions (42.0% vs. 49.4%, χ^2^(2, N=298) =1.610, p=0.205).
