# Supplementary material for: Testing the decoy effect to increase interest in colorectal cancer screening
Source: PLoS One. 2019 Mar 26;14(3):e0213668. doi: 10.1371/journal.pone.0213668 (PMC6435152; doi:10.1371/journal.pone.0213668)
Supplement: S3 Fig — (DOCX) [file pone.0213668.s010.docx]

# *S3 Figure:* Mean percentage of seeking more information with standard deviation error bars in Study 2

|  | | | | | | | | | | | |
| --- | --- | --- | --- | --- | --- | --- | --- | --- | --- | --- | --- |
|  | Control  (N=308) | | Weak decoy (Travel)  (N=160) | | Weak decoy (Wait)  (N=138) | | Strong decoy  (N=297) | | Overall  (N=903) | | p-value |
| No, not interested | 208 | (67.5%) | 100 | (62.5%) | 94 | (68.1%) | 189 | (63.6%) | 591 | (65.5%) | 0.558 |
| Yes, interested | 100 | (32.5%) | 60 | (37.5%) | 44 | (31.9%) | 108 | (36.4%) | 312 | (34.5%) |  |

*p-value refers to Chi-Square test of independence.

There are no statistically significant differences between the two weak decoy conditions (31.9% vs. 37.5%, χ^2^(2, N=298) =1.029, p=0.310).
